# Dynamic spatial dispersion of repolarization is present in regions critical for ischemic ventricular tachycardia ablation

**DOI:** 10.1016/j.hroo.2021.05.003

**Published:** 2021-05-11

**Authors:** Neil T. Srinivasan, Jason Garcia, Richard J. Schilling, Syed Ahsan, Ross J. Hunter, Martin Lowe, Anthony W. Chow, Pier D. Lambiase

**Affiliations:** ∗ Department of Cardiac Electrophysiology, The Essex Cardiothoracic Centre, Basildon, Essex, United Kingdom; † Department of Cardiac Electrophysiology, The Barts Heart Center, St Bartholomew’s Hospital, London, United Kingdom; ‡ Institute of Cardiovascular Science, University College London, London, United Kingdom; § Circulatory Health Research Group, Medical Technology Research Centre, School of Medicine, Anglia Ruskin University, Essex, United Kingdom

**Keywords:** Ablation, Dispersion of repolarization, Late potentials, Substrate mapping, Ventricular tachycardia, Ventricular repolarization, Ventricular repolarization mapping

## Abstract

**Background:**

The presence of dynamic substrate changes may facilitate functional block and reentry in ventricular tachycardia (VT).

**Objective:**

We aimed to study dynamic ventricular repolarization changes in critical regions of the VT circuit during sensed single extrastimulus pacing known as the Sense Protocol (SP).

**Methods:**

Twenty patients (aged 67 ± 9 years, 17 male) underwent VT ablation. A bipolar voltage map was obtained during sinus rhythm (SR) and right ventricular SP pacing at 20 ms above ventricular effective refractory period. Ventricular repolarization maps were constructed. Ventricular repolarization time (RT) was calculated from unipolar electrogram T waves, using the Wyatt method, as the dV/dt_max_ of the unipolar T wave. Entrainment or pace mapping confirmed critical sites for ablation.

**Results:**

The median global repolarization range (max-min RT per patient) was 166 ms (interquartile range [IQR] 143–181 ms) during SR mapping vs 208 ms (IQR 182–234) during SP mapping (*P* = .0003 vs intrinsic rhythm). Regions of late potentials (LP) had a longer RT during SP mapping compared to regions without LP (mean 394 ± 40 ms vs 342 ± 25 ms, *P* < .001). In paired regions of normal myocardium there was no significant spatial dispersion of repolarization (SDR)/10 mm^2^ during SP mapping vs SR mapping (SDR 11 ± 6 ms vs 10 ± 6 ms, *P* = .54). SDR/10 mm^2^ was greater in critical areas of the VT circuit during SP mapping 63 ± 29 ms vs SR mapping 16 ± 9 ms (*P* < .001).

**Conclusion:**

Ventricular repolarization is prolonged in regions of LP and increases dynamically, resulting in dynamic SDR in critical areas of the VT circuit. These dynamic substrate changes may be an important factor that facilitates VT circuits.

## Graphical abstract

**Figure:**
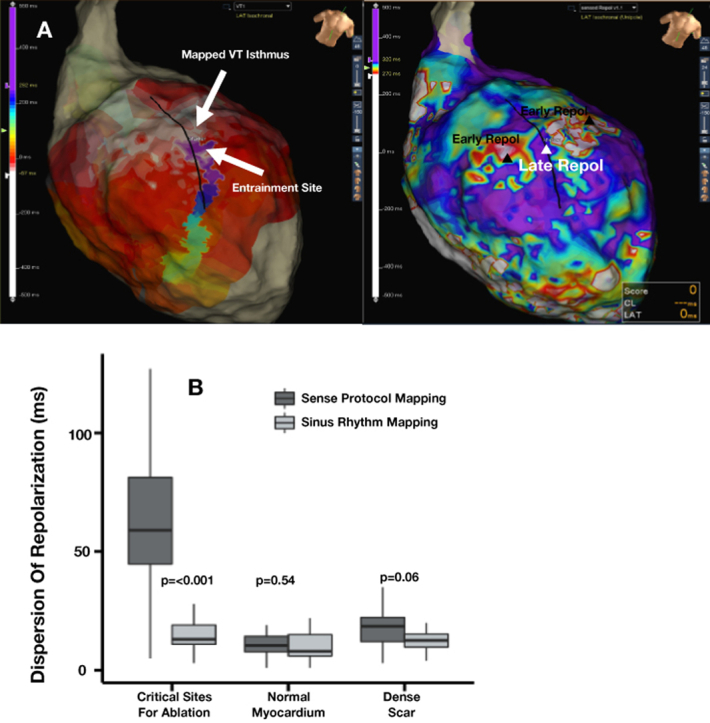

## Introduction

Ventricular tachycardia (VT) in the context of ischemic heart disease is commonly due to a reentrant arrhythmia using a fixed anatomical structure. Classically, myocardial scar acts as an anatomical substrate, containing within it channels of slow electrical conduction that facilitate reentry. The components of these channels are often dynamic and functional in nature. Josephson et al.^1^ documented the functional nature of VT circuits, with decremental delay in late potentials that are critical for VT circuit. Indeed, several methods have been developed to map functional components of the VT circuit,^2^, ^3^, ^4^ and data from initial studies suggest good outcomes when these functional regions are targeted.

Dynamic changes in conduction and repolarization within this substrate may form a critical aspect of the tachycardia mechanism when conduction velocity slows dynamically and tissue refractory periods lengthen. We have previously demonstrated dynamic prolongation of repolarization time (RT) within myocardial scar regardless of pathology,^5^ and we have also demonstrated the role of spatial dispersion of repolarization (SDR) in facilitating critical aspects of the VT circuit, with the potential association between regions of prolonged RT and late potentials (LP).^5^ Furthermore we have demonstrated the relationship between regions of distal short RT and nearby prolonged conduction time leading to unidirectional block and reentry.^6^^,^^7^ This reentry vulnerability index predicted sites of VT origin.^6^^,^^7^ Recently we have developed an automated method of mapping ventricular substrate through single extrastimulus pacing,^8^ which invokes dynamic functional LP behavior in sites critical to the VT circuit. The mechanisms for this functional behavior are unclear, but they may represent regions of repolarization delay or inhomogeneity, as we have previously demonstrated.^5^

In this study we aimed to study dynamic changes in ventricular RT, in relation to critical sites for VT ablation, using high-resolution mapping of the entire ventricle with the HD Grid (Abbott, Inc, Minneapolis), during short-coupled single extrastimuli from the right ventricular (RV) apex (“Sense Protocol”),^8^ designed to invoke conduction and total repolarization delay. We hypothesized that regions of late repolarization and SDR would correlate with the critical regions for ablation. We used the properties of the mapping system to automatically annotate repolarization maps.

## Methods

### Patient demographics

Twenty patients (mean age 67 ± 9 years, 17 male) with ischemic heart disease who were undergoing clinical VT ablation for symptomatic antitachycardia pacing, symptomatic sustained VT, or implantable cardioverter-defibrillator shocks were enrolled. Mean left ventricular ejection fraction was 26% ± 11%. The study was approved by our local ethics committee and conformed to the Declaration of Helsinki. All patients gave informed consent. Patient demographics are shown in Table 1. Not all patients had their antiarrhythmics stopped prior to procedure. Though antiarrhythmic drugs can affect both conduction time and RT, the data are paired with comparisons made intra-patient, the intrinsic rhythm being the control and the SP being the change. Thus this factor is controlled for within the data.

**Table 1:** Patient demographics

Sex	Age, y	EF, %	HTN	Stroke	CKD	Diabetes	Beta-blocker	Amiodarone	2 AAD	Etiology	Re-do
M	61	28	Y	N	Y	Y	Y	Y	Y	IHD	N
F	53	34	N	N	Y	Y	Y	N	N	IHD	N
M	84	20	Y	Y	Y	Y	Y	Y	Y	IHD	Y
M	66	20	Y	N	Y	Y	Y	N	N	IHD	Y
M	69	20	Y	N	N	N	Y	N	N	IHD	N
M	66	22	Y	N	N	N	Y	N	N	IHD	N
M	61	32	Y	N	N	Y	Y	Y	Y	IHD	N
M	74	20	Y	N	N	Y	Y	Y	Y	IHD	N
M	62	60	Y	N	Y	Y	Y	N	N	IHD	N
M	55	24	N	N	N	N	Y	N	N	IHD	N
M	61	20	N	N	N	Y	Y	Y	Y	IHD	N
M	77	12	Y	N	Y	Y	Y	Y	Y	IHD	Y
F	81	36	Y	N	N	N	Y	N	N	IHD	N
M	51	38	Y	Y	N	Y	Y	Y	Y	IHD	N
M	76	15	Y	N	N	N	N	Y	N	IHD	N
M	64	23	Y	Y	N	Y	Y	N	N	IHD	N
M	72	10	Y	N	N	N	Y	Y	Y	IHD	N
M	72	22	Y	N	N	Y	Y	Y	Y	IHD	N
M	71	27	T	N	N	N	Y	Y	Y	IHD	N
M	71	33	Y	N	N	Y	Y	Y	Y	IHD	N

AAD = antiarrhythmic drugs; CKD = chronic kidney disease; EF = ejection fraction; HTN = hypertension; IHD = ischemic heart disease.

### VT substrate mapping – The “Sense Protocol”

**Figure 1:**
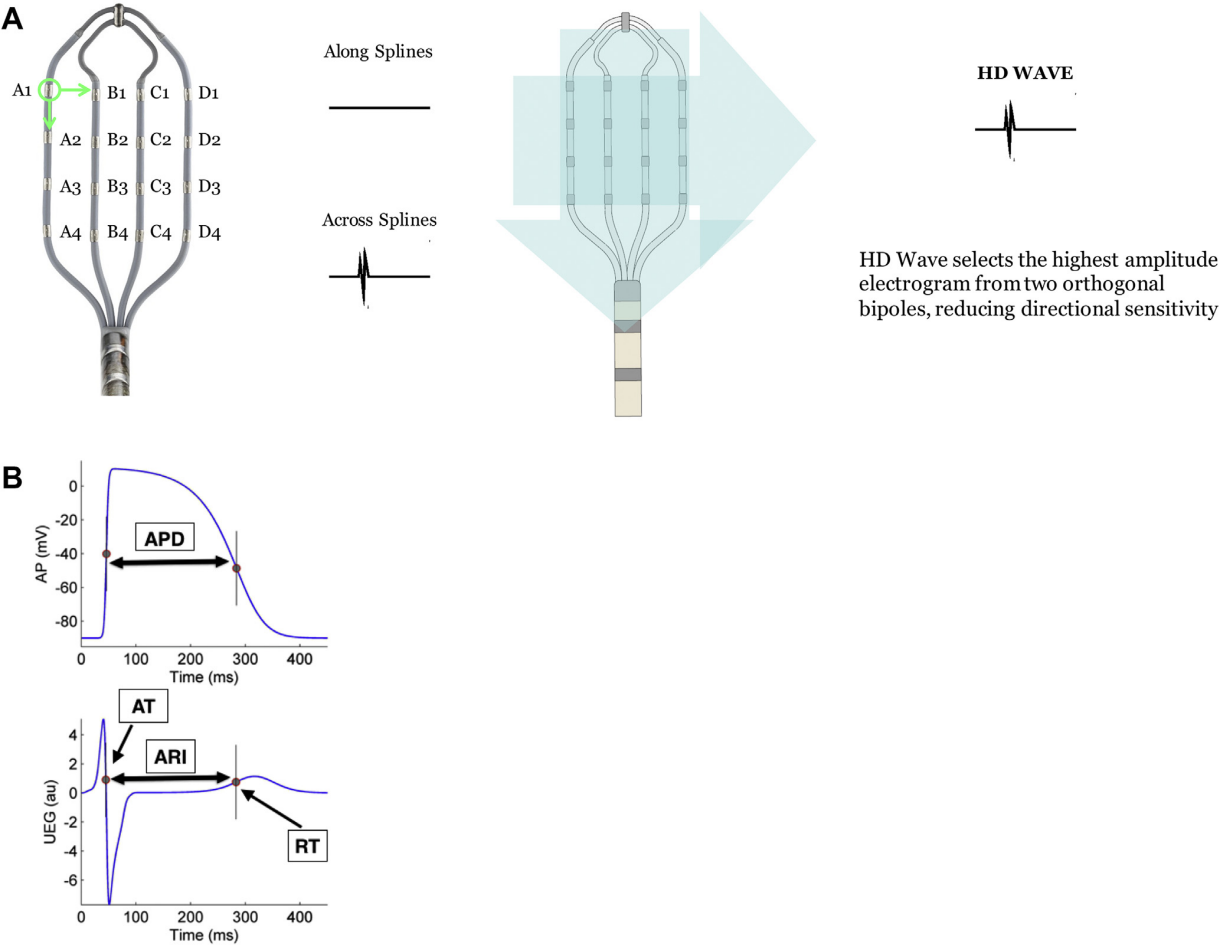
**A:** High-density (HD) grid and schematic of the HD wave solution. The HD grid consists of 16 equally spaced electrodes arranged in a 4 × 4 grid. Bipolar wavefronts are measured both along and across the splines with the HD wave solution selecting the highest-amplitude signal from 2 orthogonal bipoles, thus obviating the problem of bipolar blindness whereby a wavefront traveling along the splines would record a low-amplitude signal. **B:** Schematic of the Wyatt method used to measure repolarization time (RT) from the unipolar contact electrogram (UEG) from the HD grid. The Wyatt method measures ventricular depolarization time as dV/dt_max_ along the upslope of the unipolar electrogram T wave regardless of polarity (upright or inverted). Repolarization time (RT) is measured as the dV/dt_max_ of the unipolar intracardiac electrogram T wave. AP = action potential; APD = action potential duration; ARI = activation recovery interval; AT = activation time.

VT substrate maps were acquired with the EnSite Precision™ mapping system (Abbott, Inc) and the Advisor™ HD Grid (Abbott, Inc) (Figure 1A), which is a multipolar mapping catheter containing 16 equally spaced electrodes in a 4 × 4 grid layout (Figure 1A). A hexapolar catheter was placed in the RV apex for pacing with the proximal pole located in the inferior vena cava blood pool to reference for unipolar signals. Substrate maps were obtained during sinus rhythm (SR) and mapping the ectopic paced beat of a single sensed extrastimulus from the RV apex (Sense Protocol [SP]), as previously described,^8^ to invoke LV conduction and repolarization changes. The SP involves finding the effective refractory period (ERP) of the single paced RV sensed extra and then mapping single sensed extras at 20 ms above this interval to create a substrate map of this paced beat. This SP beat is applied every fifth interval, in order to enable the tissue to return to resting state. The SR and SP maps are made simultaneously using the Turbomap™ feature of the mapping system. The median mapping time was 46 minutes.

Bipolar voltage maps were collected using the HD wave mapping technology (Abbott, Inc), whereby bipolar recording along and across splines is enabled, with the system analyzing orthogonal bipolar wavefronts and recording the largest voltage of the 2 signals (Figure 1A). The system uses the best duplicate algorithm whereby the highest-amplitude data are collected and displayed on the map.

Normal myocardium was defined as tissue with a bipolar voltage >1.5 mV, dense scar was defined as a bipolar voltage <0.5 mV, and scar border zone was defined as a bipolar voltage 0.5–1.5 mV, consistent with previously published data.^9^ Critical sites for ablation were defined as sites of best entrainment or pace maps. Activation and entrainment mapping was performed if hemodynamically tolerated (14 patients). Where pace mapping was used we aimed for a >96% match to the clinical VT.

### Assessment of repolarization time and correlation of spatial dispersion of repolarization to regions of the heart

Total RT maps of SR and SP were made during creation of the ventricular substrate/geometry maps. Local RT was defined using the Wyatt method (Figure 1B) as the maximum first derivative of the unipolar T wave (dV/dt_max_)^10^ using existing automated software functionality within the mapping system, with manual offline checking for accuracy. The Wyatt method results in RT moments lying along the upslope of the unipolar T wave regardless of its polarity. Data from simultaneous recordings of monophasic action potentials and unipolar electrogram T waves suggest that dV/dt_max_ of the unipolar T wave corresponds to local RT.^10^ Though debate exists about its accuracy, this is the most widely published and accepted methodology to noninvasively measure RT.^1^^,^^2^^,^^3^^,^^6^ Furthermore, we have demonstrated that the Wyatt method provides a reliable approximation of local ERP in the intact human heart.^11^ In this study we did not compare the Wyatt method with ERP, owing to concerns regarding clinical risk of repetitive pacing to patients. Unipolar electrograms were filtered at 0.05–40 Hz to retain the T wave component and remove high-frequency artifacts, as previously described.^5^, ^11^, ^12^ Signals with signal-to-noise ratio <13 dB were not included in the analysis, as were signals with a flat T wave where dV/dt_max_ was not recordable. SDR was measured as the largest difference in RT in a 10 mm^2^ region, by subtracting the RT of an individual geometry point with the neighboring shortest RT point in a 10 mm^2^ radius.

Following creation of ventricular substrate maps, programmed electrical stimulation was performed to induce VT. Where the VT was hemodynamically stable, activation and entrainment mapping was used to confirm critical sites of the VT circuit that would be targeted for clinical ablation. Where VT was not tolerated, pace mapping was performed to delineate the VT exit for ablation. The sites of best pace map or entrainment were defined as critical sites for ablation. SDR was assessed at sites critical for ablation, in normal myocardium, and within dense scar during both SR and SP, to assess the dynamic effects of SP to SDR.

### Statistical analysis

Continuous variables are represented as mean ± standard deviation if normally distributed and median (25th–75th quantile) if not normally distributed. The paired Student *t* test was used to compare differences in RT between intrinsic rhythm and the SP and in different substrate characteristics if normally distributed, and Wilcoxon signed rank test was used if not normally distributed. SDR was measured as the largest difference in RT in a 10 mm^2^ region at critical sites for ablation, at dense scar, and in normal myocardium based on voltage criteria stated above. A *P* value of <0.05 was considered statistically significant. Analysis was performed using R statistical software.

## Results

### Comparison of global ventricular repolarization characteristics – Intrinsic rhythm vs Sense Protocol

A mean number of 1452 geometry points were collected per patient during intrinsic rhythm and 1384 points during the SP per patient. These were manually checked for accuracy of Wyatt method annotation of the T-wave upslope RT marker. In total, 26,139 points were analyzed in intrinsic rhythm and 24,918 points during SP across the 20 patients. Where VTs were tolerated, activation maps were created, and these were complemented by entrainment mapping to confirm circuits, or when activation maps were incomplete. Entrainment mapping was performed according to established criteria,^13^^,^^14^ looking specifically for presystolic potentials (<70% of VT cycle length). Where mapping/entrainment in VT was not possible, a pace map strategy was used (6 of 20 patients); we aimed for a match >96% to the clinical VT, as previously described.^15^ A total of 24 VTs were mapped/entrained (n = 16) or pace mapped (n = 8).

During intrinsic rhythm the global ventricular repolarization pattern within each patient was largely homogenous, with a narrow repolarization range. The mean minimum and maximum RT were 230 ± 42 ms and 395 ± 45 ms, respectively, while the median repolarization range (max-min RT per patient) was 166 ms (interquartile range 143–181 ms). During SP mapping significant SDR and maximal repolarization delay was invoked; the mean minimum RT was 219 ± 46 ms (*P* = 0.4 vs intrinsic rhythm) and the mean maximum RT was 427 ± 37 ms (*P* = .002 vs intrinsic rhythm) across all 20 patients. The median repolarization range during SP mapping was 208 ms (interquartile range 182–234 ms) (*P* = .0003 vs intrinsic rhythm). Thus significant intrapatient total RT delay was invoked during the SP.

**Figure 2:**
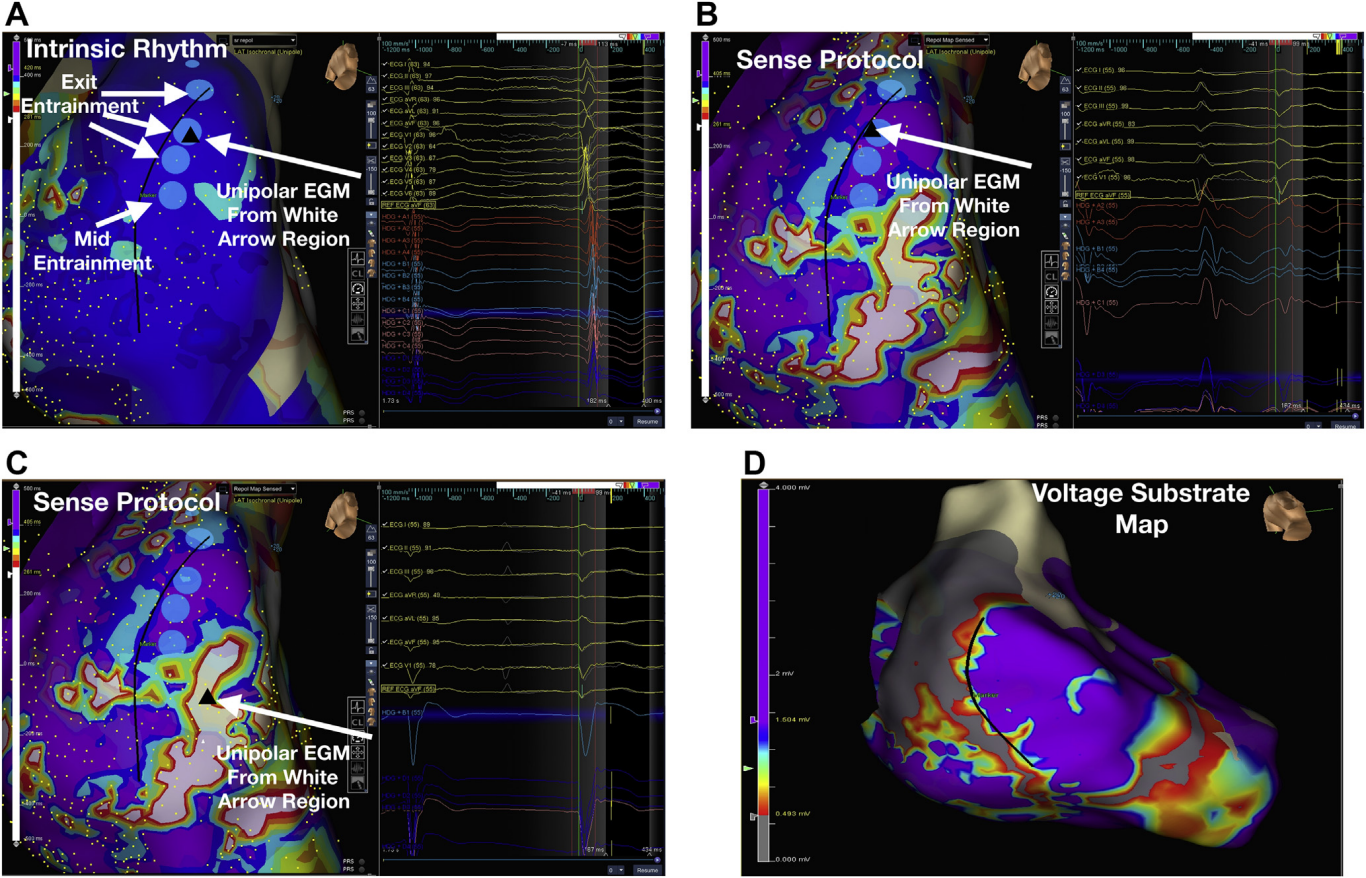
Unmasking of repolarization delay and repolarization heterogeneity via the sensed protocol in a patient with posterior/septal basal ventricular tachycardia (VT). Blue dots represent the sites of best diastolic entrainment and the black line marks the circuit with superior exit of VT. Black triangles highlight regions from which the unipolar electrogram (EGM) on the right of each map was sampled. Repolarization time (RT) color maps are shown. **A:** During intrinsic rhythm the repolarization time along the diastolic pathway is homogenous (highlighted measured RT 400 ms). **B:** During “Sense Protocol” mapping there is late ventricular repolarization in the diastolic pathway of the VT with highlighted unipolar signal (yellow triangle), showing an RT of 434 ms in a region bounded by shorter repolarization times (**C**), which line the diastolic pathway (RT 187 ms in highlighted signal region). **D:** Bipolar voltage map showing VT circuit using a scar border zone region. RT is shown on the color bar to the left of each map, and yellow markers on EGM show the annotated RT based on the Wyatt method.

Figure 2 shows an example of RT variation during intrinsic rhythm (Figure 2A), where RT is largely homogeneous compared with SP mapping (Figure 2B), where significant RT heterogeneity is seen along the diastolic pathway of the VT (Figure 2B and 2C). Figure 2D shows the bipolar voltage map of the region of the VT; it can be seen that the region of healthy tissue (purple) appropriately shortened RT in response to SP mapping, whereas the scar and scar border zone lengthened RT (Figure 2B and 2C).

### Relationship between bipolar voltage/late potentials and ventricular repolarization with dynamic single extrastimuli

**Figure 3:**
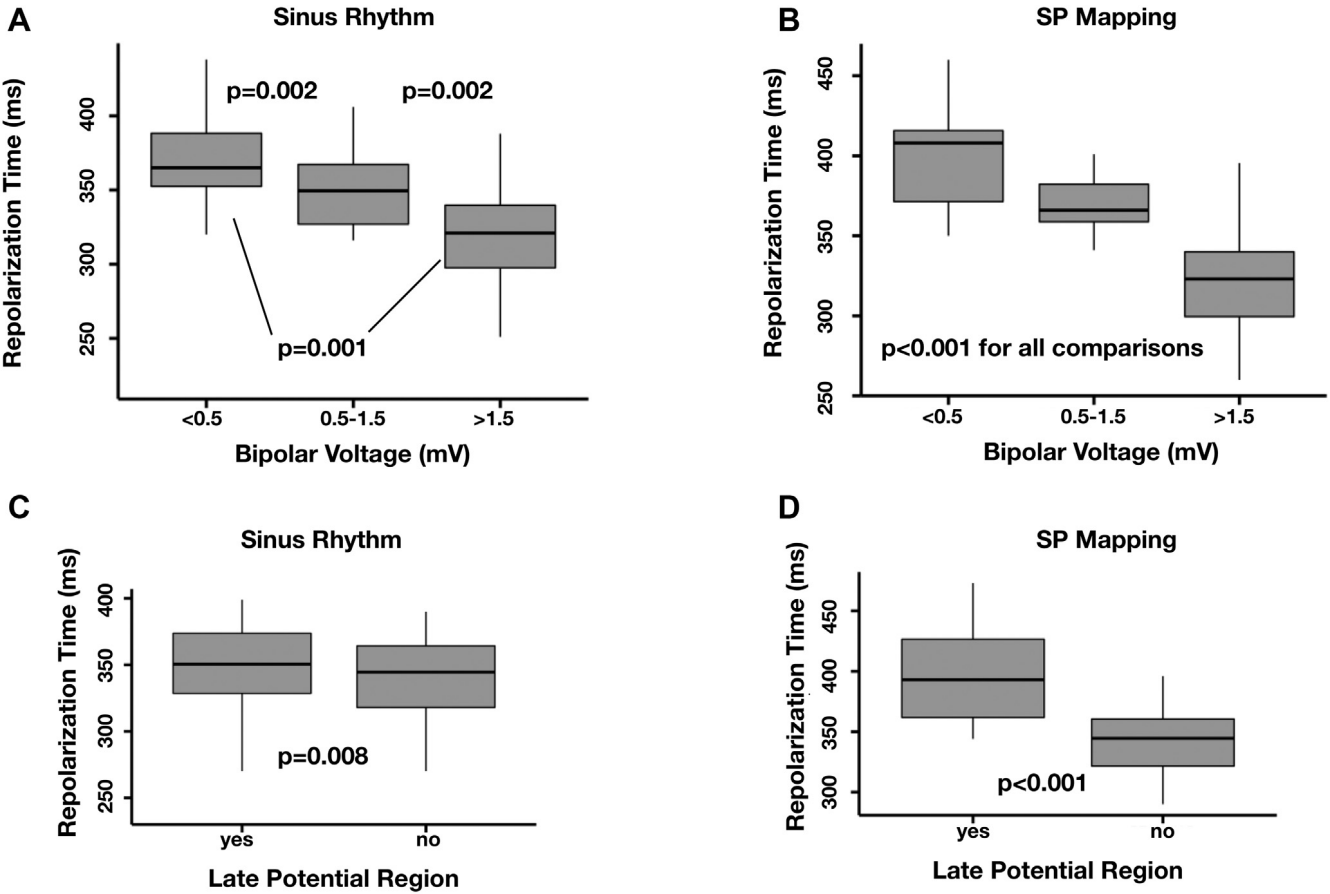
**A,B:** The relationship between ventricular repolarization time and bipolar voltage during sinus rhythm (**A**) and Sense Protocol (SP) mapping (**B**). **C,D:** Ventricular repolarization time within and outside regions of late potentials during sinus rhythm (**C**) and SP mapping (**D**).

Regions of scar and LP had longer RT than regions of healthy tissue, but this feature was exaggerated by SP mapping. Regions with bipolar voltage <0.5 mV had a higher mean RT during SR mapping (361 ± 41 ms), vs regions with voltage 0.5–1.5 mV (342 ± 42 ms, *P* = .002), vs regions with voltage >1.5 mV (317 ± 31 ms, *P* < .001 and *P* = .002, respectively), as demonstrated in Figure 3A. Regions of LP also had a longer RT during SR mapping compared to regions without LP (mean 350 ± 40 ms vs 345 ± 40 ms), *P* = 0.008, as demonstrated in Figure 3C.

During SP mapping these changes were exaggerated within regions of bipolar voltage <0.5 mV, which had a higher mean RT (402 ± 36 ms), vs regions with voltage 0.5–1.5 mV (367 ± 30 ms), vs regions with voltage >1.5 (321 ± 3 ms), *P* < .001 for all 3 comparisons, as demonstrated in Figure 3B. Regions of LP also had significantly longer RT during SP mapping compared to regions without LP (mean 394 ± 40 ms vs 342 ± 25 ms), *P* < .001, as demonstrated in Figure 3D.

### Functional areas of repolarization dispersion relate to critical areas of the VT circuit

**Figure 4:**
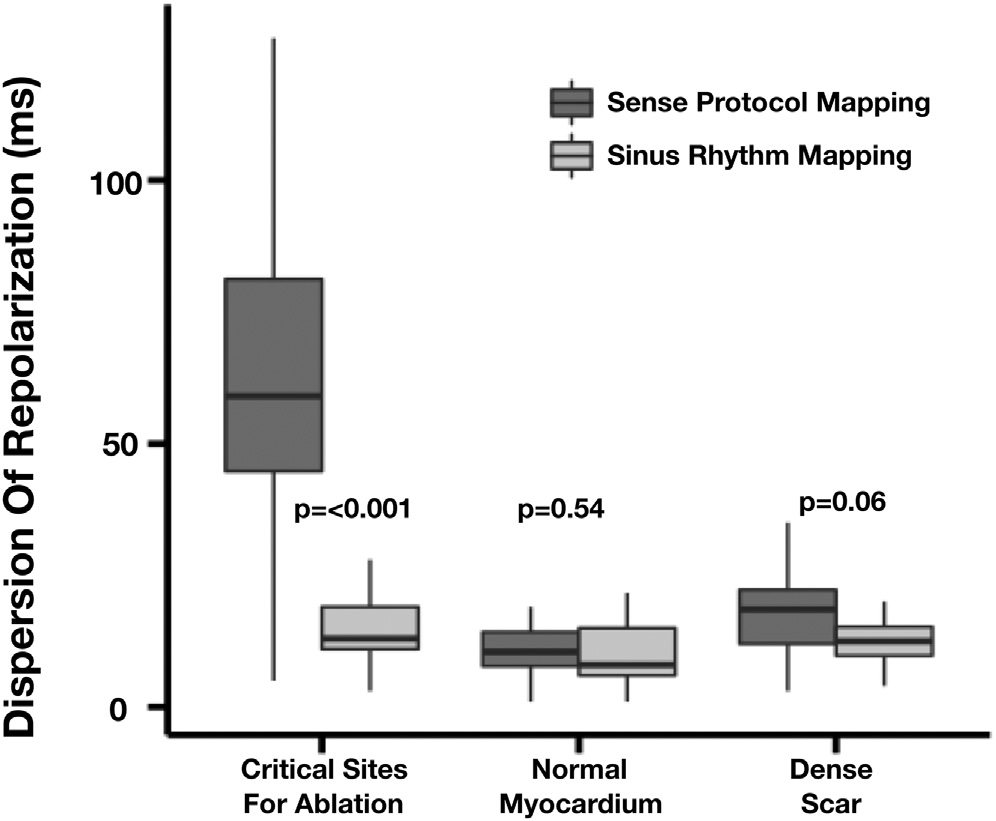
Dispersion of repolarization at sites critical for ventricular tachycardia ablation in regions of normal myocardium and dense scar based on voltage criteria, during Sense Protocol mapping (*dark gray*) and sinus rhythm mapping (*light gray*).

SDR/10 mm^2^ was greater in critical sites of the VT circuit during SP mapping 63 ± 29 ms vs SR mapping 16 ± 9 ms (*P* < .001), suggesting dynamic intrapatient SDR in critical regions (Figure 4, left). In paired regions of dense scar (Figure 4, right) there was no significant difference in largest measured SDR between SP mapping (18 ± 8 ms) and SR mapping(13 ± 4 ms) (*P* = 0.06), suggesting this was not a global feature of scar tissue (Figure 4, middle), while in paired regions of normal myocardium (Figure 4, middle) there was no significant alteration in SDR during SP mapping vs SR mapping (SDR 11 ± 6 ms vs 10 ± 6 ms, *P* = .54).

**Figure 5:**
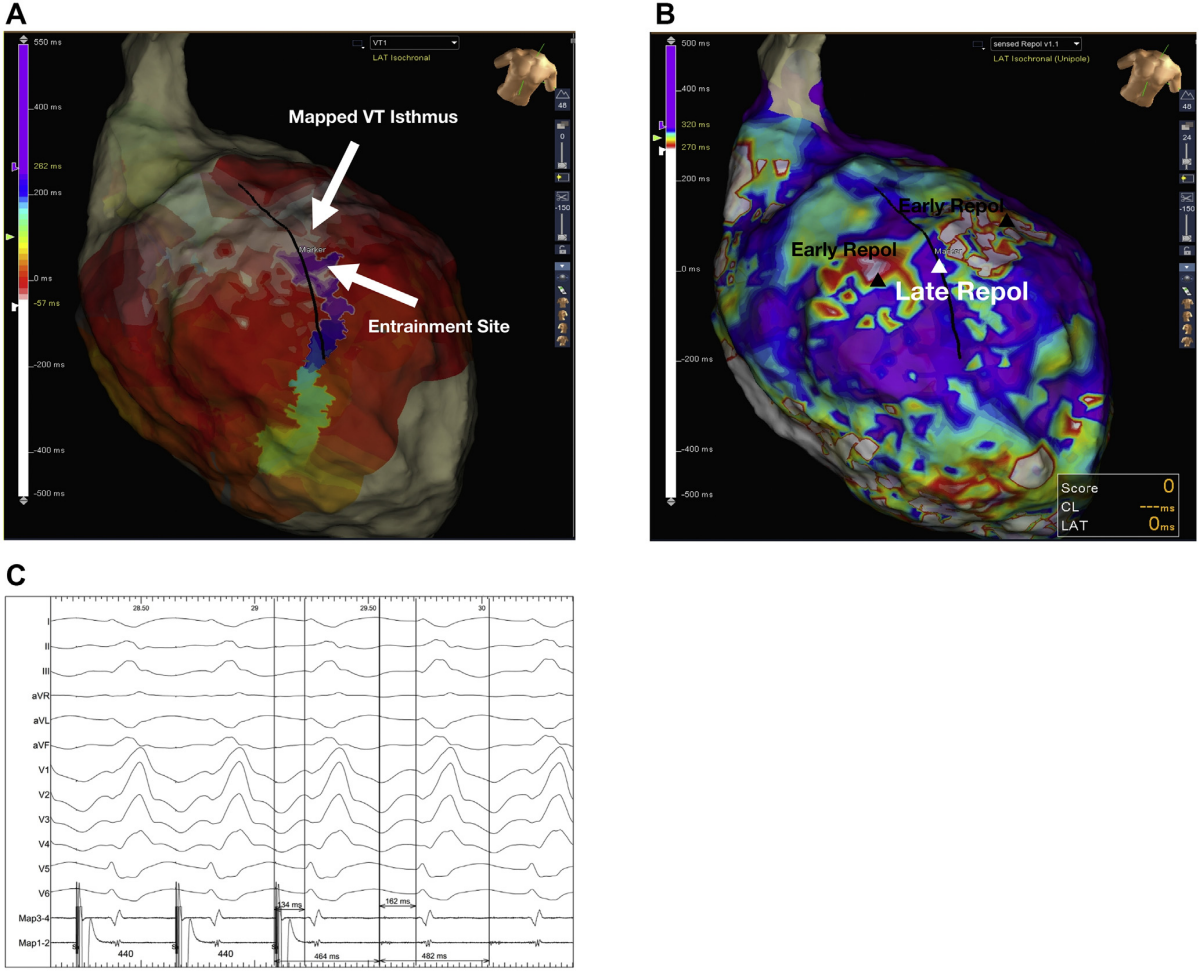
Example of ventricular tachycardia (VT) circuit relating to repolarization. **A:** VT wavefront is shown, corresponding to the late ventricular repolarization (**B**) wavefront during Sense Protocol. **B:** The late repolarization corridor corresponded to an area of concealed entrainment (**C**) and is bounded by early repolarization on either side. Black line represents VT circuit.

**Figure 6:**
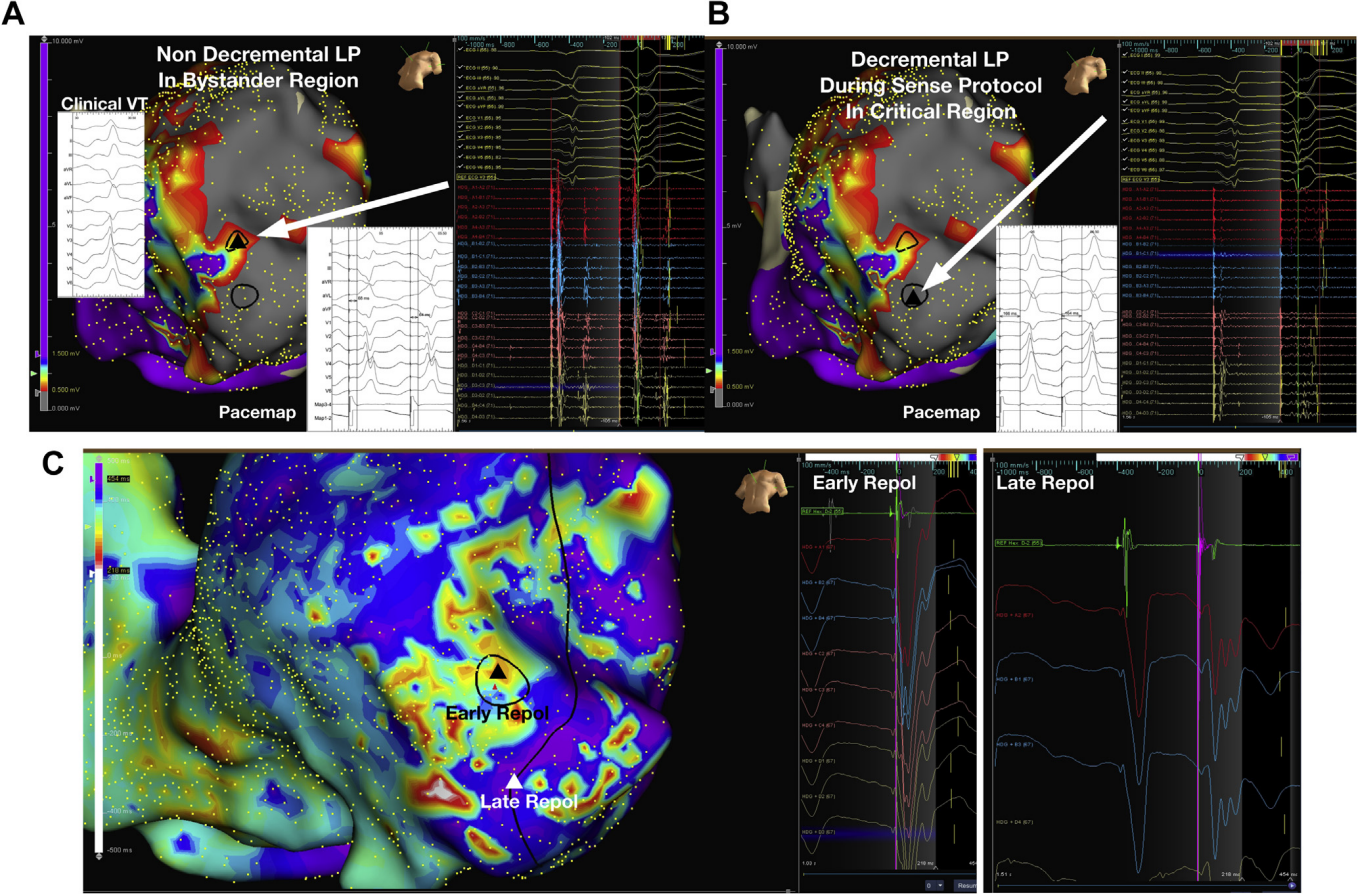
Voltage substrate map in a patient with apical and anterior scar. **A:** Black triangle highlights an area on the scar border zone with late potentials (LP) shown on the signals to the right. These do not delay on Sense Protocol single extrastimuli. *Insets*: Clinical ventricular tachycardia (VT) and pace map (lower right), which does not match the clinical VT in this nondecremental LP area. **B:** Triangle shows a region within the scar where there are hidden LP and LP delay on the electrograms of the HD grid to the right. Pace map in this region (*inset*) shows a good correlation to clinical VT with stimulus to electrogram time of 166 ms. **C:** Repolarization color map of the same region is shown, with early repolarization in region from panel A, and late repolarization in region from panel B. The black line marks the course of the clinical VT with superior exit along a region of late repolarization bounded by early repolarization either side. Unipolar electrograms with repolarization time markers are displayed in the electrograms in panel C. Despite LP in both regions, the unipolar T waves are of a different polarity in these nearby regions, resulting in spatial dispersion of repolarization.

Figure 5 illustrates an example in a patient where regions of late repolarization (Figure 5B) are bordered by regions of shorter repolarization along critical regions of the VT circuit/isthmus (Figure 5A), creating islands of repolarization dispersion, which may serve to facilitate VT. Figure 6 shows a region within dense ventricular scar where there are decremental (Figure 6A) and nondecremental LP (Figure 6B) during SP mapping. The region of decremental LP correlated with a good pace map (Figure 6B), and regions of late repolarization (Figure 6C) and dynamic RT change in response to SP.

## Discussion

This study uses a new high-density mapping catheter; Advisor HD Grid, and a unique pacing protocol (“Sense Protocol”)^8^ to identify the repolarization characteristics of critical substrate for VT ablation. Our main findings are as follows: (1) Dynamic single extrastimuli result in increased maximal RT and global dispersion of repolarization. (2) Ventricular RT is longer in regions of low voltage and this feature is exaggerated by single extrastimuli. (3) Significant spatial dispersion of repolarization is present over small areas of tissue in critical regions of the VT circuit during single extrastimulus SP mapping, highlighting that repolarization changes in these critical regions are dynamic and may facilitate the occurrence of VT.

### Repolarization time lengthens with single extrastimuli and in regions of low voltage

Activation mapping of VT remains a challenge in patients in whom VT is poorly tolerated, not inducible, or nonsustained. Conventional substrate mapping also has limitations and new methods are required to improve outcomes, which are often poor.^16^ The majority of ventricular substrate mapping is performed during SR, but substrate changes may be dynamic.^8^ We have previously demonstrated homogenous regional repolarization in the normal human ventricle.^12^ In the structurally abnormal ventricle we have shown prolonged action potential duration (APD) and RT in regions of scar,^5^ in a limited study using decapolar catheters in isolated regions of the ventricle. Here, we demonstrate prolonged ventricular repolarization in regions of scar/low voltage using a multipolar grid catheter with high-density total global ventricular repolarization mapping both in intrinsic rhythm and with the SP.

We demonstrate that maximal RT and global ventricular repolarization dispersion are dynamic and increase in response to RV single extrastimuli (SP mapping). Additionally we show that RT increases as bipolar voltage decreases, and this is again exaggerated by SP mapping. Prolonged RT and increased global dispersion of repolarization have previously been demonstrated in heart failure models,^17^ but never in the intact human heart.

Ventricular repolarization involves a complex interaction between a series of electrical currents. Studies have shown that failing myocytes demonstrate upregulation of Cx43,^8^ calcium overload, and downregulation of potassium currents,^16^^,^^18^ all serving to prolong ventricular repolarization, perhaps to improve contractility. This feature may be further emphasized during short coupled extrastimuli, where rate adaptation is blunted in failing myocytes. In response to short R-R intervals ventricular repolarization usually shortens, an adaptive feature that is largely regulated by calcium handling and sodium channels,^19^^,^^20^ which may fail to adapt in diseased tissue owing to calcium overload and the failure of the late sodium current to recover.

### Dynamic spatial dispersion of repolarization is present in critical regions of the VT circuit and may facilitate the occurrence of VT

It is well documented that VT detected on device traces are most frequently related to extrasystolic activity,^21^ suggesting dynamic substrate changes in response to ectopics as a trigger for VT initiation. In view of this we developed the SP, which studies the tissue in its most maladapted form in response to single extrastimuli, as opposed to repeated stimuli, where APD shortening and adaptation occurs.^8^ Although repolarization prolongation was seen in regions of LP and low voltage during SR mapping, within regions critical for the VT circuit SDR/10 mm^2^ was only 16 ± 9 ms compared to 10 ± 6 ms in regions of normal voltage, suggesting that despite the adaptive substrate changes regional repolarization is relatively homogenized to prevent arrhythmia and achieve coordinated electromechanical coupling.

During SP mapping, SDR increased significantly to 63 ± 29 ms / 10 mm^2^ within critical regions of the VT circuit, suggesting that this dynamic change may play an important role in regions that facilitate VT. This increase in SDR during SP mapping vs SR mapping was unique to critical regions and not present globally within low or normalvoltage tissue (Figure 4), suggesting that failure to maintain repolarization homogeneity may create the dynamic substrate that facilitates VT. This may be due to a combination of conduction and APD delay in critically diseased tissue that fails to adapt to extrastimuli, promoting regions of conduction block through heterogeneous regional refractory properties, and may facilitate reentry. The promotion of conduction block through increased SDR and RT may allow late entry of the electrical wavefront into the scar, scar border zone, or regions of VT channels, which can then reactivate healthy tissue, which has short RT. This concept may be explained by the coupling between RT and activation time; we have previously demonstrated a loss of repolarization coupling between regions of early activation and late activation in the intact human heart, and these data support this in patients with ischemic cardiomyopathy (Figure 6).^12^

Because we were unable to separate activation time from RT and therefore delineate APD in the mapping system, it is unclear whether dispersion of activation/conduction plays a greater role than dispersion of APD/total RT in facilitating VT in critical regions. This is a feature that needs further refinement within mapping systems, in order to subtract these time points. It is interesting that the greatest SDR was seen in regions of LP, suggesting conduction delay plays a major role. Further studies are needed to delineate this in greater detail, to assess whether RT maps add further information to improve VT ablation. However, our previous work^6^ suggests that both SRD and dispersion of activation time play an important role.

In animal studies it has been shown that local repolarization gradients of >3.2 ms/mm create source sink environments that facilitate the formation of unidirectional block^22^ and that abrupt changes in repolarization regionally by 20 ms or more may facilitate the conditions for unidirectional block^23^ by facilitating conduction delay.^23^^,^^24^ Our intact human heart studies are in keeping with these data showing a 6.3 ms/mm^2^ repolarization dispersion during SP mapping in critical regions of the VT circuit; crucially, these findings were dynamic and SDR was 6 times greater during SP mapping compared to SR mapping in critical regions. These findings suggest that dynamic methods of ventricular mapping may be required to elucidate critical substrates for ablation in order to improve outcomes.

Although it is unclear whether repolarization dispersion alone may facilitate reentry,^25^ repolarization dispersion has been demonstrated as a unique feature in human heart studies of heart failure.^26^^,^^27^ In animal models, it has been shown that whereas dispersion of repolarization may identify regions susceptible to block, it does not differentiate between bidirectional block, which is usually not arrhythmogenic, and unidirectional block, which is a basic requirement for reentry.^25^ Recently a mapping protocol has been developed that aims to locate areas of potential unidirectional block.^6^^,^^7^^,^^28^ This algorithm, referred to as the reentry vulnerability index, has already shown considerable promise in the clinical setting^6^^,^^7^ and suggests that excitable gaps are small. However, the limitation of this method is the need for repetitive pacing and also the fact that it fails to compare dynamic substrate changes between intrinsic rhythm and short coupled pacing. In the present study we focus on evaluating the ability of SP-induced dynamic changes in dispersion of repolarization to identify sites critical for VT, and we demonstrate the presence of functional changes in these regions. We do not map excitable gaps in VT. These data suggest that further characterization of dynamic functional changes within the ventricle may aid identification of substrate targets for VT ablation. Further research is required to compare assessment of repolarization against depolarization/conduction delay to see if it improves catheter ablation of VT. Recent work using SP VT mapping^8^ suggests improvements in long-term outcomes of VT ablation. It remains to be seen whether ablation of SP SDR will improve these outcomes further or streamline the efficiency of mapping VT substrate.

### Limitations

Accepted voltage cut-offs for scar threshold were applied based on published data; however, debate exists as to what the correct optimal voltage cut-offs should be. Ventricular repolarization was measured using the Wyatt method; although the accuracy of this surrogate marker for RT on using the unipolar contact electrogram is debated, it remains the most accurate measure available.^10^ The purpose of the study was to perform mapping within the existing mapping system and we were not able to directly measure APD by subtracting activation time from RT within the mapping system to generate independent maps, and therefore were limited by measuring total RT only. Recording was only performed of endocardial LV electrograms, and therefore we were not able to assess transmural heterogeneities. A resolution of greater than 16 spaced electrodes may be required to map SDR across the ventricle in more detail.

## Conclusion

Ventricular repolarization is prolonged in low-voltage tissue and lengthens dynamically, resulting in dynamic dispersion of repolarization in critical areas of the VT circuit. These substrate changes may be an important factor that facilitates human VT circuits through the creation of unidirectional block. Further evaluation of these dynamic mapping methods are required to assess their effects on VT ablation and to assess whether ablating these regions can suppress VT. Additionally, the role of this technique in assessing bystander and epicardial circuits needs further assessment.

## Funding Sources

This work was supported by 10.13039/501100000765University College London Hospitals Biomedicine 10.13039/501100000272National Institute for Health Research. N.T. Srinivasan was supported by a 10.13039/501100000274British Heart Foundation Clinical Research Training Fellowship (10.13039/100006959FS/14/9/30407). P.D. Lambiase was supported by the 10.13039/501100000265Medical Research Council (G0901819), Barts BRC and Stephen Lyness Research Fund.

## Disclosures

The following authors have received speaker fees from Abbott in the last 10 years: Neil Srinivasan, Anthony Chow, Martin Lowe, Richard Schilling, and Pier Lambiase. Pier Lambiase receives research grants from Boston Scientific & Abbott. All remaining authors have declared no conflicts of interest

## Authorship

All authors attest they meet the current ICMJE criteria for authorship.

## Patient Consent

All patients provided written informed consent.

## Ethics Statement

The study complies with the guidelines set forth in the Declaration of Helsinki. The local ethics authority approved the study.

**Key Findings**

▪ Sensed single extrastimuli result in increased maximal repolarization time and global spatial dispersion of repolarization.
▪ Ventricular repolarization time is longer in regions of low voltage and this feature is exaggerated by sensed single extrastimuli.
▪ Significant spatial dispersion of repolarization is present over small areas of tissue in critical regions of the ventricular tachycardia (VT) circuit during single extra mapping that are not present during sinus rhythm mapping.
▪ Dynamic repolarization changes are present in patients with VT and may facilitate the occurrence of VT.
